# Salinity, pH and temperature growth ranges of *Halophytophthora* isolates suggest their physiological adaptations to mangrove environments

**DOI:** 10.1080/21501203.2020.1714768

**Published:** 2020-01-14

**Authors:** Chun-Jui Su, Sung-Yuan Hsieh, Michael Wai-Lun Chiang, Ka-Lai Pang

**Affiliations:** a Institute of Marine Biology and Centre of Excellence for the Oceans, National Taiwan Ocean University, Keelung, Taiwan; b Bioresource Collection and Research Center, Food Industry Research and Development Institute, Hsinchu, Taiwan; c Department of Chemistry, City University of Hong Kong, Hong Kong SAR

**Keywords:** Eicosapentaenoic acid, marine fungi, oomycota, physiology, unsaturated fatty acid

## Abstract

Species of *Halophytophthora* are early colonisers of fallen mangrove leaves in the tropics but recently found commonly in temperate areas. In mangrove habitats, temperature and salinity change rapidly daily (high/low tide) and seasonally (summer/winter, rainy/dry seasons). Mangrove organisms have to develop adaptive strategies to thrive in such a physiologically challenging environment. In this study, growth of three isolates of *Halophytophthora avicenniae* and two isolates of *H. batemanensis* was tested under combined effects of 3 temperatures (15°C, 25°C, 37°C), 3 pHs (6, 7, 8) and 4 salinities (4 ‰, 8 ‰, 16 ‰, 32 ‰). No/little growth was observed at 37°C and growth saturation occurred earlier at 25°C than at 15°C. The log phase of growth was steeper at pH 6 than pH 7 and 8. Temperature and pH were found to exert a greater effect on growth than salinity. Generally, a reduction of growth rate was observed at pH 8 and 15°C. Increase in salinity caused a slight decrease in growth, most noticeable at 32 ‰. The wide growth ranges of temperature, salinity and pH of *Halophytophthora* isolates suggest that they are well adapted to the physical and chemical conditions of mangrove habitats.

## 1. Introduction

The genus *Halophytophthora* is classified under the Pythiales, Oomycota (www.mycobank.com). Characterisation of species of *Halophytophthora* is mainly based on their asexual reproductive characteristics: sympodial or irregular sporangiophore branching, papillate/non-papillate zoosporangia with smooth wall or spines, with or without operculum, vesicle and plug (Nakagiri 2002).

*Halophytophthora* currently includes 14 marine and freshwater species: *H. avicenniae, H. bahamensis, H. batemanensis, H. elongata, H. epistomia, H. exoprolifera, H. fluviatilis, H. insularis, H. masteri, H. mycoparasitica, H. polymorphica, H. porrigovesica, H. souzae* and *H. vesicula*, after several phylogenetic studies of the group using various genes (Hulvey et al. 2010; Marano et al. 2014a, 2014b; Li et al. 2016; Bennett et al. 2017; Jesus et al. 2019). *Halophytophthora* is polyphyletic (Marano et al. 2014b) and consequently, some species have been transferred to other known/novel genera (e.g. *H. kandeliae* to *Phytopythium*, Marano et al. 2014a) while some await further studies (e.g. *H. exoprolifera*, Marano et al. 2014b). *Halophytophthora sensu stricto* includes *H. avicenniae, H. batemanensis, H. insularis, H. polymorphica, H. souzae* and *H. vesicula* (Jesus et al. 2019).

*Halophytophthora* can be isolated from freshwater to saline waters, from fallen mangrove leaves to seagrasses and from tropical (e.g. Brazil, Hong Kong, Japan, Philippines, Taiwan) to temperate (e.g. Denmark, France, Germany, Netherlands) regions, although most have been reported from fallen leaves of mangroves in tropical/subtropical locations (Leaño et al. 2000; Nakagiri 2000; Nigrelli and Thines 2013; Pang et al. 2015; Jesus et al. 2019; Man in ’t Veld et al. 2019; Nale et al. 2019). In the above studies, species composition of *Halophytophthora* was found to be different between different salinity gradients, geographical zones and substrates (hosts). Seasonal variation (i.e. warm/cold seasons) and substrate specificity (i.e. *Bruguiera gymnorrhiza*/*Rhizophora stylosa*) were also found in *Halophytophthora* species isolated from the mangrove of Iriomote Island (Nakagiri 2000). In mangrove environments, *Halophytophthora* species are early colonisers of fallen leaves and fulfill a degrader role in mangroves by mineralising leafy materials for nutrition by the production of enzymes, such as cellulases in *H. vesicula* (Raghukumar et al. 1994). Zoospores of *Halophytophthora* are potential food resources for grazing and filter-feeding zooplankton and metazoan invertebrates (Sime-Ngando et al. 2011). Recently, an unknown *Halophytophthora* species was found to inhibit germination of seeds of *Zostera marina* in North Atlantic and Mediterranean (Govers et al. 2016, 2017).

Mangroves are a harsh habitat as it is known to be a sink of pollutants and their physical (temperature) and chemical (salinity and pH of seawater) conditions change daily and seasonally. *Halophytophthora* species were found to tolerate low concentrations (<10 ppm) of Cu(II), Zn(II) and Pb(II) but high concentrations (>100 ppm) impaired both growth and reproduction (Leaño and Pang 2010). Leaño et al. (2000) investigated individual effects of pH, temperature and salinity on growth and sporulation of selected *Halophytophthora* species and found that they had wide growth and sporulation ranges. However, these factors influence growth of *Halophytophthora* concurrently in mangrove environments and therefore, this paper investigated the combined effects of temperature, pH and salinity on growth of *Halophytophthora* isolates cultured from fallen leaves collected in mangroves of Taiwan. Specifically, this paper reported mycelial growth of three isolates of *Halophytophthora avicenniae* and two isolates of *H. batemanensis* under combined effects of 3 temperatures (15°C, 25°C, 37°C), 3 pHs (6, 7, 8) and 4 salinities (4 ‰, 8 ‰, 16 ‰, 32 ‰).

## 2. Materials and methods

### 2.1. Isolation

Fallen leaves were collected on different dates near the river mouth at She-zi, Chu-nan, Pu-zi and Sih-cao mangroves, Taiwan (Table 1). The leaves were washed with tap water to remove surface mud and rinsed twice with sterile seawater (30 ‰). Washed leaf pieces (~1 cm^2^ square) were cut from the leaves and inoculated in 15 ml sterile seawater (30 ‰) supplemented with 0.5 g/L each of Penicillin G sodium salt and Streptomycin sulphate in Petri dishes and incubated at 25°C in the dark for 2 days. Individual zoosporangia were picked under a stereomicroscope with a flame-sterilised tweezer and sub-cultured onto peptone-yeast extract-glucose seawater agar plates (PYGS: 4 g/L glucose, 4 g/L peptone, 4 g/L yeast extract, 14 g/L agar, 30 g/L artificial sea salt) supplemented with antibiotics (0.5 g/L Penicillin G-sodium salt and 0.5 g/L Streptomycin sulphate) as pure cultures.

**Table 1.** Origin of the test *Halophytophthora* isolates and their identification.

			GenBank accession no.		BLASTn result (identity/sequence length (bp)/coverage/similarity)	
Species	Isolate no.	Mangrove site	18S rDNA	ITS rDNA	18S rDNA	ITS rDNA
*Halophytophthora avicenniae*	IMB157	She-zi	KM205193	KM205202	Identified in Pang et al. (2015)	
*Halophytophthora avicenniae*	IMB215	Chu-nan	MN559555	MN565895	HQ161104/1705/100%/100%	KM205202/835/100%/99.87%
*Halophytophthora avicenniae*	IMB225	Pu-zi	MN559565	MN565898	HQ161104/1705/100%/100%	KM205202/835/100%/99.88%
*Halophytophthora batemanensis*	IMB217	Sih-cao	MN559557	MN565900	GU994179/1737/100%/99.8%	GU258916/914/100%/96.33%
*Halophytophthora batemanensis*	IMB221	Pu-zi	MN559561	MN565903	GU994179/1737/100%/100%	GU258916/914/100%/99.37%

### 2.2. Identification

One isolate (IMB157) was identified previously while the rest was identified based on a nucleotide BLAST search of their 18S and internal transcribed spacer regions (ITS) of the rDNA in National Centre for Biotechnology Information (NCBI). Mycelia were scraped from the surface of PYGS plates and ground into fine powder in liquid nitrogen using a mortar and a pestle. Genomic DNA was extracted using the DNeasy Plant DNA Extraction Kit (Qiagen) according to the manufacturer’s instructions. The nuclear rRNA genes were amplified using primers NS1/NS4 or NS6 (18S) and ITS1 or ITS5/ITS4 (ITS) (White et al. 1990). PCR reactions were performed in a 25 µL volume containing 0.5 μl of the extracted DNA, 1 μl each of the two primers (10 μM), 12.5 μl Taq premix (BIOMAN, Taipei), 10 μl PCR water. The amplification cycle consisted of an initial denaturation step of 95°C for 5 min followed by 35 cycles of (a) denaturation (95°C for 30 s), (b) annealing (55°C for 30s) and (c) elongation (72°C for 30 s) and a final 5 min elongation step at 72°C. The PCR products were analysed by agarose gel electrophoresis and sent to Genomics, Taipei, Taiwan for sequencing using the same primers. The sequences obtained were checked for ambiguity and assembled in MEGA7 (Kumar et al. 2016) and submitted to NCBI for a nucleotide BLAST search.

### 2.3. Production of zoospores

The isolates were inoculated onto vegetable juice agar plates [20% commercial vegetable juice (Bomy daily 100% fruit & vegetable juice, Taiwan), 0.2% CaCO_3_, 12 g/L agar, 2.5% seawater] at 25°C in darkness for 1 week. Five agar disks were cut from the growing edge of the colony and inoculated into an empty Petri dish containing 2.5% sterile seawater at 25°C in darkness for 24 h. This zoospore suspension (zoospores released into the seawater) was adjusted to 1.1 × 10^3^ spores/ml for all isolates and used as the inoculum.

### 2.4. Growth study

Mycelial growth of the *Halophytophthora* isolates under various combinations of temperatures, pHs and salinities were measured based on a microtitre plate (Co-Star 3595, Corning, Maine, USA) method designed by Langvad (1999). A medium containing 4 g/L glucose, 4 g/L yeast extract and 4 g/L peptone was used as the growth medium and 180 μl of this medium adjusted to various pHs (6, 7, 8) and salinities (4 ‰, 8 ‰, 16 ‰, 32 ‰ made by sea salt) were dispensed into wells of the microtitre plate. Acidity/alkalinity of the medium was adjusted using hydrochloric acid (Taiwan Green Version Technology Ltd., New Taipei City, Taiwan) and sodium hydroxide (Panrea, Barcelona, Spain). The spore suspensions (20 μl) were added to the wells and mixed briefly. For each treatment, four replicates (wells) were done. The inoculated plates were incubated at 15°C, 25°C and 37°C. A set of control wells for each treatment, i.e. the growth medium with no spores, was also prepared. Absorbance of the wells of the microtitre plates was measured daily at 630 nm using the multi-detection microplate readers (Synergy HT, BioTek) for 9 days. Growth of the isolates was represented by subtraction of the absorbance of the inoculated wells to that of the control wells.

### 2.5. Statistical analysis

For growth curve fitting, the sigmoidal function, logistic I, had the best fit for the data and was used. The modelling was performed using the program Origin 9.1 (OriginLab Corporation, USA). This function is a generalised logistic model and has the formula:

(1) $$y=\frac{a}{1+e^{-k\left(x-x_{c}\right)}}$$

where, y represents the observed optical density at time x; a, the upper asymptote; xc, the point of inflection on the x-axis; k, growth rate constant; e, the base of the natural logarithm.

For comparison of growth between different temperatures, one-way ANOVA was performed using SPSS 25 (IBM Software, Inc., America) using Tukey-test with post hoc comparison based on the average of absorbances of the last three incubation days at stationary phase or the absorbance on the last incubation day when growth did not reach stationary phase. The same test was also run for pH and salinity.

## 3. Results

**Figure 1.**
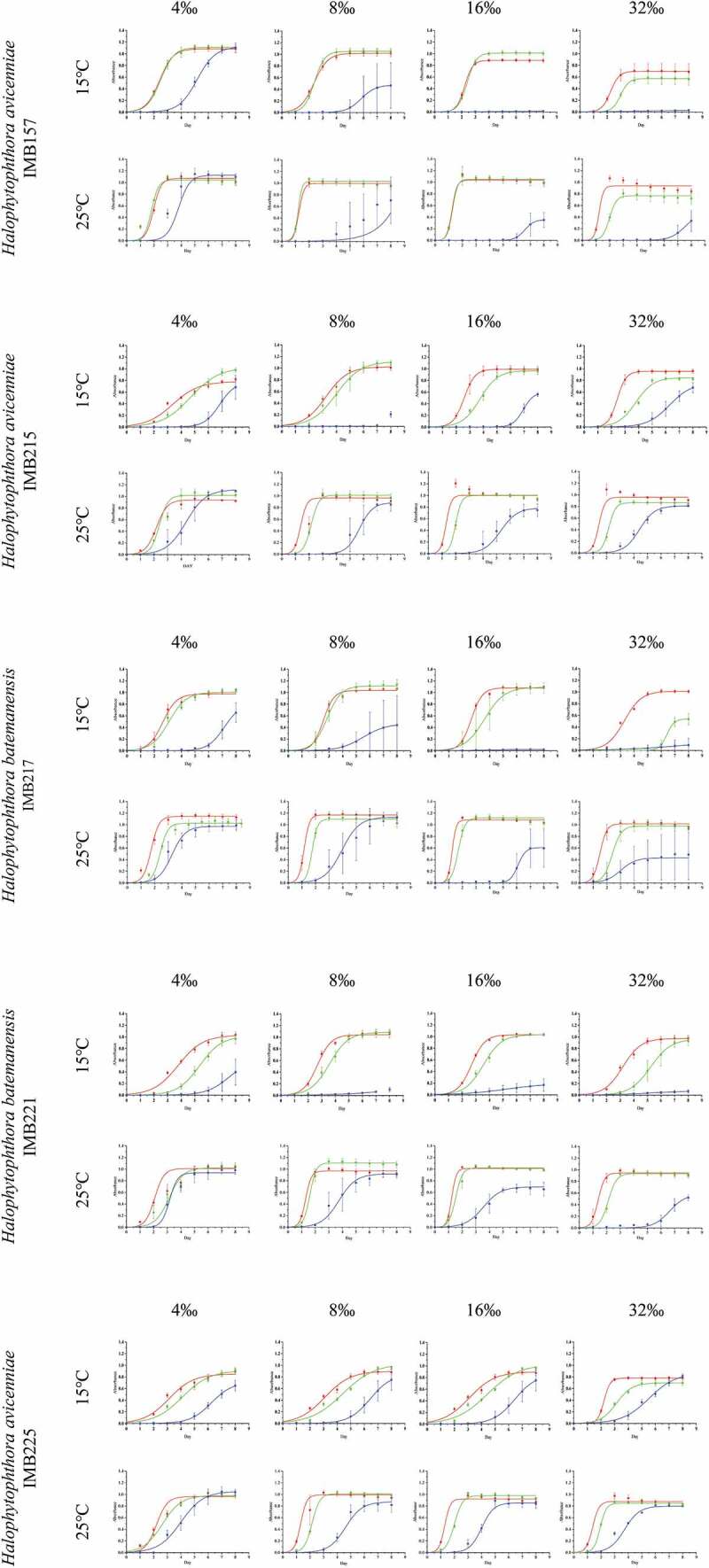
Growth curves of five *Halophytophthora* isolates under two temperatures (15°C, 25°C), three pHs (6: red line, 7: green line, 8: blue line) and four salinities (4 ‰, 8 ‰, 16 ‰, 32 ‰) based on fitting of the generalised logistic model, logistic I.

Figure 1 shows the growth curves of the test isolates of *Halophytophthora avicenniae* and *H. batemanensis* under different incubation temperatures (15°C, 25°C), pHs (6, 7, 8) and salinities (4 ‰, 8 ‰, 16 ‰, 32 ‰) under the model logistic I. No/little growth was observed at 37°C for all isolates and the available growth data points at this temperature did not fit in any growth models. At 25°C, growth reached saturation on Day 3 to 4 for pH 6 and pH 7 for all salinities while the time to reach growth saturation was beyond Day 4 when incubated at 15°C. The log phase of growth for pH 6 was the steepest while that for pH 8 was the least steep. The effect of salinity on growth of the *Halophytophthora* isolates was not apparent, except at 32 ‰ where a slower growth rate at pH 7 and pH 8 was observed. For *H. avicenniae*, isolate IMB157 grew faster than IMB215 and IMB225; growth responses of the two isolates of *H. batemanensis* were similar.

**Figure 2.**
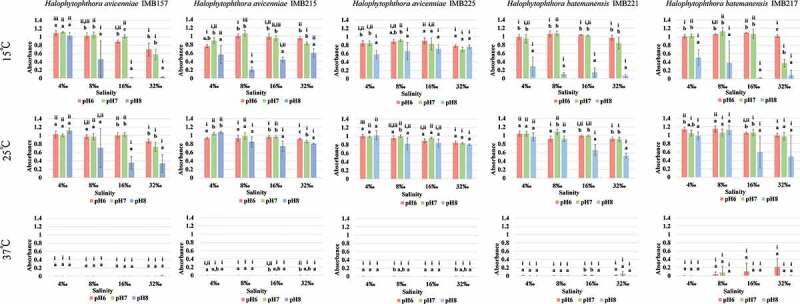
Growth of five *Halophytophthora* isolates under three temperatures (15°C, 25°C, 37°C), three pHs (6, 7, 8) and four salinities (4 ‰, 8 ‰, 16 ‰, 32 ‰) based on the average of the absorbance of the last three incubation days at the stationary phase of growth or the absorbance on the last incubation day when growth did not reach the stationary phase. The bars with the same letter or Roman numeral represent no significant difference (*p*< 0.05).

Figure 2 shows comparisons of growth at different pHs and salinities at 15°C, 25°C and 37°C for each isolate based on the average absorbance of growth on Day 8, 9 and 10 (stationary phase). Temperature and pH exerted pronounced growth effects on the isolates, when compared with salinity. At 37°C, little growth was observed for the two isolates *H. batemanensis* at pH 6 and pH 7 and >8‰ salinity while no noticeable growth was detected for *H. avicenniae*. Generally, growth at different pHs and salinities was similar at both 15°C and 25°C, except for pH 8. Reduced growth was noted at pH 8. Better growth was observed for pH 8 at 25°C than at 15°C; at the same pH, the three *H. avicenniae* isolates had better growth than the two *H. batemanensis* isolates. Increase in salinity only caused a slight decrease in growth, and it was most notable at 32 ‰ salinity.

## 4. Discussion

Mangroves are a physiologically challenging environment for organisms with their significant variations in salinity (e.g. rainy/dry seasons, high/low tide) and temperature (e.g. summer/winter, lower temperature when water comes in during high tide on sunny days/higher temperature when the sun heats up mangrove floor during low tide) daily and seasonally. As degraders of leaf litter in mangrove environments, the selected isolates of *Halophytophthora avicenniae* and *H. batemanensis* showed a wide tolerance of salinity and temperature for growth, as well as a wide pH growth range, suggesting that they can physiologically adapt to changes in physical (temperature) and chemical (pH and salinity of seawater) conditions. The results are in agreement with Leaño et al. (2000) who reported that different *Halophytophthora* species showed wide growth ranges of pH (6–9), salinity (0–60 ‰) and temperature (10–35°C), although better growth was observed at neutral pH, 10–40 ‰ salinity and 20–30°C.

No/little growth was observed at 37°C for the *Halophytophthora* isolates. Jesus et al. (2019) found that the two new species of *Halophytophthora* (*H. souzae* and *H. insularis*) did not grow at 40°C but were able to resume growth after reintroduction to 21°C, suggesting that 40°C was not a lethal temperature for these species. Growth resumption at a lower temperature after incubation at 37°C was not examined in this study but this ability expressed by *Halophytophthora* species confirms their adaptation to temperature fluctuations of mangrove habitats.

The *Halophytophthora* isolates showed growth at 15°C, suggesting that this genus can colonise colder climate. *Halophytophthora* isolates (within *Halophytophthora sensu stricto*) were cultured from leaf litter/organic debris at sites near Hamburg, Germany and were able to grow from 5°C to 30°C although the optimal temperatures for growth among the isolates varied (Nigrelli and Thines 2013). Unknown *Halophytophthora* species were also isolated from the Wadden Sea (Denmark), the Dutch Delta area (the Netherlands) and Thau lagoon (France), suggesting that species of this genus are not restricted to tropical/subtropical locations but have a much wider worldwide distribution than previously thought (Man in ’t Veld et al. 2019). Hassett et al. (2019), using a high throughput sequencing technique, detected sequence signatures of *Halophytophthora* in the Arctic. Distribution of *Halophytophthora* and related taxa worldwide might have been overlooked; alternatively, *Halophytophthora* species can disperse through recent events, e.g. through ballast water (Nigrelli and Thines 2013; Pang et al. 2013).

Optimal growth pH for *Halophytophthora* species was found to be at neutral or slightly acidic pH (Leaño et al. 2000; this study), although average pH of seawater is around pH 8. The sediment pH of mangroves of Taiwan where fallen leaves rest on during low tide (at the time when they were collected) was found to be slightly acidic (Hseu and Chen 2000; Hsueh and Lee 2000). Combustion of fossil fuel has already caused a reduction of ocean pH by 0.1 unit and the prediction model suggested that a further pH reduction of 0.7 units would be resulted if carbon dioxide continues to be released (Caldeira and Wickett 2003). The optimal growth of *Halophytophthora* species at neutral/acidic pH suggests that ocean acidification might not exert a strong effect on mangrove *Halophytophthora*. In a microcosm study of seawater of the North Sea, a higher abundance and an increase in species richness of marine fungi were observed after incubation at pH 7.81 and 7.67 compared with the normal pH of the North Sea (8.10), showing that marine filamentous fungi and yeasts might benefit from seawater acidification (Krause et al. 2013a, 2013b).

The results of this study may have industrial implications. *Halophytophthora sensu lato* species (including *Salispina spinosa*) are known to produce both saturated and unsaturated fatty acids in their mycelia, especially the essential polyunsaturated arachidonic and eicosapentaenoic acids, among others (Pang et al. 2015). The fast growth rate, i.e. 3–4 days reaching the stationary phase of growth, for most species under optimal conditions may be advantageous for industrial production of these beneficial fatty acids.

In conclusion, the test isolates of *H. avicenniae* and *H. batemanensis* showed wide growth ranges of temperature (15°C, 25°C), pH (6, 7, 8) and salinity (4 ‰, 8 ‰, 16 ‰, 32 ‰). The optimal conditions for growth were 25°C, pH 6 and 4–16 ‰ salinity. These physiological characteristics enable *Halophytophthora* species to cope with the fluctuating chemical (pH/salinity of seawater) and physical (air/seawater temperature) conditions and to be one of the dominant colonisers of fallen leaves in mangrove environments. However, the effects of these environmental stressors on sporulation and zoospore germination of *Halophytophthora* are unknown and this area requires further studies.
